# Tackling selective neuronal vulnerability: Subnuclei involvement of amygdala‐predominant Lewy body disease in early‐onset Alzheimer’s disease

**DOI:** 10.1002/alz.093381

**Published:** 2025-01-03

**Authors:** D. Luke Fischer, Lawren VandeVrede, William W. Seeley, Salvatore Spina, Fattin Wekselman, Argentina Lario Lago, Lea T. Grinberg

**Affiliations:** ^1^ Memory and Aging Center, UCSF Weill Institute for Neurosciences, University of California San Francisco, San Francisco, CA USA; ^2^ Memory & Aging Center, Department of Neurology, University of California in San Francisco, San Francisco, CA USA; ^3^ Department of Pathology, University of California San Francisco, San Francisco, CA USA

## Abstract

**Background:**

Lewy body disease (LBD) often co‐occurs with Alzheimer’s (AD), resulting in more significant cognitive decline than AD or LBD alone. LBD’s hallmarks, asyn‐positive Lewy bodies and neurites, propagate from the enteric system or olfactory bulb to the amygdala, which acts as a gatekeeper for spread to other structures. Initially, LBD appears in the central or cortical nuclei, reflecting brainstem or olfactory origins. A third pattern of LBD is quite prevalent in sporadic and familial early‐onset AD (EOAD). This amygdala‐predominant LBD (AP‐LBD) type does not conform with the most used staging systems for LBD and has received little attention. We found a link between asyn seeding‐competent fibrils in cerebrospinal fluid (CSF) and LBD’s transition from the amygdala, supporting its gatekeeper role. The factors enabling asyn propagation in the amygdala remain unknown. Our goal is to map asyn and tau markers in EOAD cases with permissive vs. non‐permissive AP‐LBD in amygdala subnuclei, identifying spread pathways and vulnerable cell populations.

**Method:**

We identified all postmortem EOAD cases with AP‐LBD from the UCSF Neurodegenerative Disease Brain Bank (n = 47), 14 of which have banked CSF for asyn seeding amplification testing. We are using multiplex immunofluorescence to label: phosphorylated asyn; phosphorylated, mis‐conformed, or truncated tau; and all neurons. Markers will be quantified from digital pathology scans, and we will analyze the degree of colocalization by region for the central nucleus, basolateral nuclei, and cortical nucleus.

**Result:**

Pending analysis, we will report the pattern of LBD across subnuclei. Of eight CSF samples, 37.5% were positive for asyn seeding with six samples pending. Asyn and tau markers by subnucleus will be compared between CSF‐positive and ‐negative cases.

**Conclusion:**

Mapping AP‐LBD across amygdala subnuclei may yield insights into selectively vulnerable neurons. This research has the potential to enhance our understanding of AP‐LBD and its underlying mechanisms, ultimately contributing to the development of more targeted diagnostic and therapeutic strategies in the realm of neurodegenerative diseases.

